# Potential roles of gut microbes in biotransformation of natural products: An overview

**DOI:** 10.3389/fmicb.2022.956378

**Published:** 2022-09-29

**Authors:** Yucui Zhao, Xinqin Zhong, Junyuan Yan, Congying Sun, Xin Zhao, Xiaoying Wang

**Affiliations:** ^1^Ministry of Education Key Laboratory of Pharmacology of Traditional Chinese Medical Formulae, Tianjin University of Traditional Chinese Medicine, Tianjin, China; ^2^School of Chinese Materia Medica, Tianjin University of Traditional Chinese Medicine, Tianjin, China; ^3^State Key Laboratory of Component-based Chinese Medicine, Tianjin University of Traditional Chinese Medicine, Tianjin, China

**Keywords:** natural products, gut microbes, enzyme system, biotransformation, bioavailability

## Abstract

Natural products have been extensively applied in clinical practice, characterized by multi-component and multi-target, many pharmacodynamic substances, complex action mechanisms, and various physiological activities. For the oral administration of natural products, the gut microbiota and clinical efficacy are closely related, but this relationship remains unclear. Gut microbes play an important role in the transformation and utilization of natural products caused by the diversity of enzyme systems. Effective components such as flavonoids, alkaloids, lignans, and phenols cannot be metabolized directly through human digestive enzymes but can be transformed by enzymes produced by gut microorganisms and then utilized. Therefore, the focus is paid to the metabolism of natural products through the gut microbiota. In the present study, we systematically reviewed the studies about gut microbiota and their effect on the biotransformation of various components of natural products and highlighted the involved common bacteria, reaction types, pharmacological actions, and research methods. This study aims to provide theoretical support for the clinical application in the prevention and treatment of diseases and provide new ideas for studying natural products based on gut biotransformation.

**Graphical Abstract:**
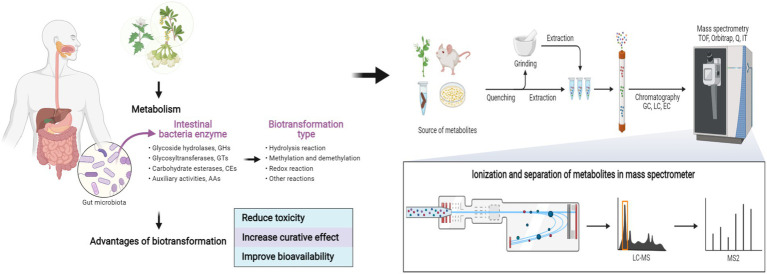
Biotransformation and metabolism of natural products based on gut microbes. Created with BioRender.com.

## Introduction

The gut microbiota is composed of 1,000–1,250 kinds of bacteria that interact with humans in various forms, such as symbiosis and parasitism, and this interaction greatly affects human health *via* microbial metabolites as signal molecules (Liu et al., 2017; de Vos et al., 2022). The gut microbes constitute a dynamic and diversified micro-ecosystem, which is a natural barrier to resisting pathogenic bacteria (Chopyk and Grakoui, 2020; Zhao and Maynard, 2022). Gut microbes have abundant enzyme systems, including glucosidase, reductase, lyase, transferase, etc., and greatly expand the metabolic response pool in the human body (Wilson and Nicholson, 2017; Fushinobu and Abou Hachem, 2021).

Natural products are small molecules produced naturally by any organism including primary and secondary metabolites.^1^ This article mainly describes natural products of plant origin, including nutrients and drugs. They easily interact with gut microbiota because of their complex components and long residence time in the gut. Generally, the residence time for exogenous substances is 1–6 h in the small intestine and 1–3 days in the colon (Chu and Traverso, 2022). Specific gut microbes decompose and transform natural products to produce rich metabolites and functional compounds with physiological activities that cannot be synthesized by the host itself (Koppel et al., 2017; Xie et al., 2020). Microbial transformation in natural products usually refers to the chemical reactions that are used to modify the structure of natural products substrates, such as hydrolysis, methylation, demethylation, redox, and cyclization reaction (Morgan et al., 2022; Rocchetti et al., 2022). Gut microbiota remarkably affects the chemical modification, pharmacological activity, and metabolic mechanism of natural products. The potential utility of gut microbes for large-scale synthesis of active metabolites and production of compounds has not been investigated. Studying these gut microbes, metabolites, and the reactions involved in the interactions between natural products and gut microbiota is of great significance in the exploration of the pharmacological mechanisms and utilization of natural products. In this review, we introduce the resident gut microbes that contribute to the transformation of natural products and summarize the transformation pathways between natural products and specific microbes classified by the reactions. Moreover, the advantages, research methods, and future directions of gut microbial in the conversion of natural products are discussed to provide a theoretical basis for the modern application of natural products and the precise treatment through gut microbiota.

## Key gut microbes in the biotransformation of natural products

**Figure 1:**
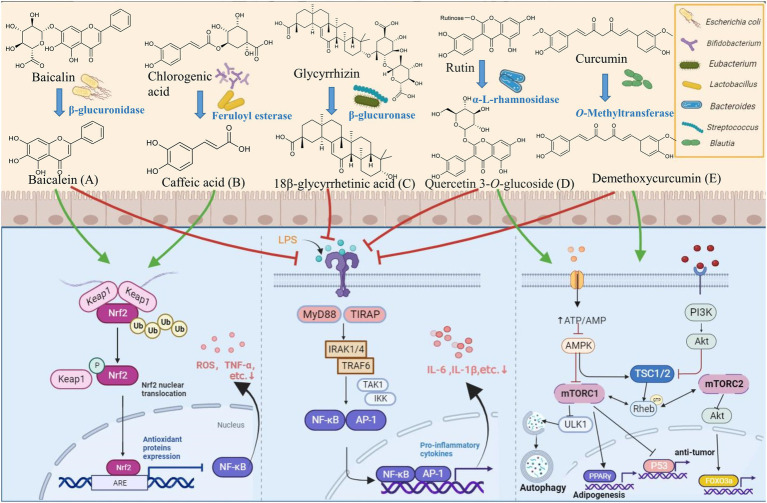
Biotransformation of natural products by key gut microbes. **(A)** β-glucuronidase of *E. coli* HGU-3 catalyzes hydrolysis of baicalin to yield baicalein (Han et al., 2016; Li D. et al., 2019). **(B)** Feruloyl esterases from *B. animalis*, *L. reuteri*, *L. helveticus*, and *L. fermentum* catalyzes hydrolysis of chlorogenic acid into caffeic acid (Raimondi et al., 2015; Pang et al., 2016; Aguirre Santos et al., 2018). **(C)** β-glucosidase of *Eubacterium* L-8 and *Streptococcus* LJ-22 catalyzes hydrolysis of glycyrrhizin to 18β-glycyrrhetinic acid (Kim et al., 1999; Chen B. et al., 2021). **(D)** α-L-rhamnosidase from *Bacteroides* sp. 45 catalyzes hydrolysis of rutin to quercetin 3-*O*-glucoside (Riva et al., 2020). **(E)** *O*-Methyltransferase from *Blautia* sp. MRG-PMF1 catalyzes the demethylation of curcumin to demethoxycurcumin (Burapan et al., 2017a,b). Created with BioRender.com.

Oral administration is the preferred route for drug delivery, and oral drugs account for 84% of the top 50 best-selling drugs in the US and European markets (Lennernäs and Abrahamsson, 2005; Vinarov et al., 2021). In recent years, the influence of gut microbiota on the stability of oral administration of natural products has received much attention. The intestinal tract has abundant bacteria that help with normal digestive function, in which about 98% of gut microbes in healthy subjects can be classified into four phyla, Firmicutes, Bacteroidetes, Proteobacteria, and Actinobacteria (Manor et al., 2020; Ye et al., 2021; de Vos et al., 2022). Some gut microbes such as *Escherichia coli*, *Bifidobacterium*, *Eubacterium*, *Lactobacillus*, *Bacteroides,* and *Streptococcus* participate in the biotransformation of natural products, and part of their metabolites are conducive to intestinal absorption and play a notable pharmacological role (Al-Ishaq et al., 2021; Augusti et al., 2021; Figure 1).

### Escherichia coli

*Escherichia coli* is a Gram-negative, spore-free, facultative anaerobic bacterium, which mainly inhabits the intestines of vertebrates (Foster-Nyarko and Pallen, 2022). Part of *E. coli* can produce glycosidase to participate in the transformation of exogenous substances, resulting in its beneficial role (Rodríguez-Daza et al., 2021; Candeliere et al., 2022). For example, *E. coli* HGU-3 produces β-glucuronidase to hydrolyze the *O*-glycosidic bond in baicalin to produce baicalein (Kim et al., 1996, 1998; Han et al., 2016). Baicalein depresses histamine-induced scratching behavior more effectively than baicalin at the same dose and presents anti-inflammatory and anti-oxidant effects by inhibiting Nrf2-ARE and NF-κB signaling pathway (Chen et al., 2000; Trinh et al., 2010; Ye et al., 2014). Some *E. coli* strains have high specific activity for curcumin conversion. Curcumin is reduced to dihydrocurcumin (DHC) and tetrahydrocurcumin (THC) by the highly expressed NADPH-dependent curcumin/dihydrocurcumin reductase (CurA) of *E. coli* DH10B, whose whole-genome sequence had already been determined (Hassaninasab et al., 2011; Tan et al., 2014). DHC and THC (20 μM) reduce triglyceride levels in OA-induced L02 and HepG2 cells by regulating mRNA and protein expression levels of SREBP-1C and PPARα and attenuate OA-induced liver adipogenesis in an AMPK-dependent manner; DHC and THC have novel therapeutic benefits over curcumin in hepatic steatosis (Chen et al., 2018; Yu et al., 2018). *E. coli* Nu, *E. coli* MC, and *E. coli* WC-1 have cinnamyl esterase activity, which can release hydroxycinnamic acids through the hydrolysis of conjugated hydroxycinnamates and free hydroxycinnamates exhibit antioxidant and anticancer properties both *in vitro* and *in vivo* (Couteau et al., 2001). At present, a good understanding of the genetic and biochemical characteristics of *E. coli* may contribute to the synthesis of natural product derivatives with various health activities *in vitro.*

### Bifidobacterium

*Bifidobacterium* is a widespread and abundant genus belonging to the phylum Actinobacteria and is among the first colonizers of gut microbiota for humans (Satti et al., 2021; He et al., 2022). The most common *Bifidobacterium* in the human gut include *B. adolescentis*, *B. angulatum*, *B. bifidum*, *B. breve*, *B. catenulatum*, *B. dentium*, *B. longum*, *B. pseudocatenulatum*, and *B. pseudolongum* (Turroni et al., 2009; Hidalgo-Cantabrana et al., 2017), accounting for <10% of the adult human microbiome, but they are linked to host health (Turroni et al., 2008). Certain species of *Bifidobacterium* can generate phenolic acids by expressing feruloyl esterase. For example, the feruloyl esterase of *B. animalis* can hydrolyze chlorogenic acid (CHA) into caffeic acid (CAA; Raimondi et al., 2015). CAA (10–30 mg/kg) can prevent acetaminophen-induced acute liver injury in mice by increasing Nrf2 transcription (Raimondi et al., 2015; Pang et al., 2016). The participation of partial *Bifidobacterium* promotes the metabolism of flavanones, glycosides, and saponins in the gut. β-glucosidase and demethylase in *B. longum* R0175 promote 3-(3′-hydroxyphenyl) propionic acid and 3-(phenyl) propionic acid production from hesperidin through ring-cleavage and demethylation (Pereira-Caro et al., 2018). *B. longum* SBT2928 hydrolyzes six major human and two animal bile salts (Tanaka et al., 2000). Thus, *Bifidobacterium* may regulate bile acid metabolism and reduce cholesterol levels *in vivo*. In addition, *B. breve* ATCC 15700 produces β-glucosidase to cleave glycoside at the C-3 and C-20 positions of ginsenoside Rd. to generate deglycosylated ginsenoside compound K (Zhong et al., 2016; Zhang R. et al., 2019). These metabolic characteristics make *Bifidobacterium* a prime candidate for the development of symbiosis to make natural products potentially beneficial.

### Eubacterium

The genus of *Eubacterium* strains is Gram-positive, which forms one of the core genera of the human gut microbiota and shows widespread colonization of the human gut (Mukherjee et al., 2020). Some *Eubacterium* species produce glycosidase, reductase, etc., and participate in the metabolism of exogenous substances (Zhang J. et al., 2019; Ellenbogen et al., 2021). *E. ramulus* is one of the most widely studied flavonoid-degrading gut bacteria, and it is prevalent in the human intestine. Chalcone isomerase and flavanone-/flavanonol-cleaving reductase from *E. ramulus* degrade certain flavonoids to produce chalcone, and dihydrochalcone (Gall et al., 2014). Dihydrochalcone and its metabolites have anti-inflammatory and antioxidant effects, which can down-regulate the secretion of pro-inflammatory cytokines in RAW 264.7 and rescue LPS-induced oxidative phosphorylation (Choi et al., 2021). Braune et al. investigated the degradation of flavonol quercetin and flavone luteolin by *E. ramulus* strain wK1 and found that resting cells and enzyme preparations convert these flavonoids into 3, 4-dihydroxyphenylacetic acid, and 3-(3, 4-dihydroxyphenyl) propionic acid *via* the reduction of 2, 3-position double bonds and subsequent ring fission (Braune et al., 2001). Phloretin hydrolase from *E. ramulus* strain wK1 hydrolytically cleaves the C-C bond, which is adjacent to the aromatic A-ring of phloretin to 3-(4-hydroxyphenyl)-propionic acid and phloroglucinol (Schoefer et al., 2004; Braune et al., 2019). *E. cellulosolvens* ATCC 43171^T^ may contribute to the deglycosylation of flavonoid *O*- and *C*-glucosides (luteolin 6-*C*-glucoside and apigenin 6-*C*-glucoside) through the fermentation of the liberated glucose portion. The deglycosylation of *C*-glucosides is exclusively catalyzed by bacterial enzymes (Braune and Blaut, 2012; Braune et al., 2016). *Eubacterium* L-8 hydrolyzed terpenoid glycyrrhizin (GL) to 18β-glycyrrhetinic acid (18β-GA; Kim et al., 2000). 18β-GA prevents OVA-induced airway allergic inflammation by inhibiting NF-κB phosphorylation and enhancing the Nrf2/HO-1 pathway (Liu et al., 2022). These metabolic transformations provide more information about the diverse array of benefits that humans derive from *Eubacterium* spp. However, further *in vivo* studies are necessary to maximize the potential benefits the *Eubacterium* genus has to offer.

### Lactobacillus

The genus *Lactobacillus* belongs to the phylum Firmicutes, which can balance the micro-community and protect gastrointestinal mucosa (Dempsey and Corr, 2022). Some *Lactobacillus* species are rich in metabolic enzymes, such as α-rhamnosidases, tannase, gallate decarboxylases, etc. and they transform exogenous substances (Reverón et al., 2017; Li B.C. et al., 2019; Ferreira-Lazarte et al., 2021). *L. rhamnosus* NCTC 10302, which has both β-glucosidase and α-rhamnosidase activities, converts hesperetin-7-*O*-rutinoside and naringenin-7-*O*-rutinoside to their respective aglycones and 3-(phenyl) propionic acid by hydrolysis, ring fission, and dehydroxylation (Pereira-Caro et al., 2018). *L. plantarum* expresses tannase to hydrolyze gallate, protocatechuate esters with a short aliphatic alcohol substituent, and complex gallic tannins to produce gallic acid (Jiménez et al., 2014). Gallic acid (11.5–46 μg/ml) plays a protective role in LPS-induced inflammation and oxidative stress by inhibiting the MAPK/NF-κB pathway and activating the Akt/AMPK/Nrf2 pathway (Tanaka et al., 2018). Fang et al. observed that gallic acid and pyrogallol are produced by the degradation of gallotannins by gallotannin-metabolizing enzymes in *L. plantarum* WCFS1. This study implies the potential role of prebiotic-probiotic interactions in the prevention of diet-induced metabolic disorders (Reverón et al., 2015; Fang et al., 2019). Daidzein is reduced to dihydrodaidzein by *Lactobacillus* sp. Niu-O16 with daidzein reductase activity (Wang et al., 2007; Heng et al., 2019). Dihydrodaidzein (2.5–5 μM) inhibits NF-κB activation and MAPK phosphorylation, thereby improving osteoporosis (Kim et al., 2019). *L. casei*, *L. plantarum*, and *L. acidophilus* highly influence the deglycosylation of piceid to resveratrol (Basholli-Salihu et al., 2016). This conversion is important for increasing the bioavailability and bioactivity of piceid. Feruloyl esterases from *L. reuteri, L. helveticus*, and *L. fermentum* hydrolyze chlorogenic acid to release caffeic acid (Aguirre Santos et al., 2018). These findings open a new perspective on the role of *Lactobacillus* in health-promoting pharmaceutical and food product applications. However, the underlying transformation mechanism deserves further study.

### Bacteroides

Members of the genus *Bacteroides* are Gram-negative obligate anaerobes, which account for 25% of the total bacteria in the colon and play multiple roles in the human gut bacteriome (Zafar and Saier, 2021). *Bacteroides* species such as *B. fragilis*, *B. distasonis*, *B. ovatus*, and *B. thetaiotaomicron* are commonly detected in the clinic (Wexler, 2007). *Bacteroidetes* spp. possesses a series of hydrolases and participates in inter-species cross-feeding relationships with their microbial neighbors by converting foreign substances (Sonnenburg et al., 2004; Zafar and Saier, 2021). *In vitro* co-incubation experiments showed that certain *Bacteroides* species are involved in the biotransformation of flavonoids. *Bacteroides* sp. 45 expresses α-L-rhamnosidase and β-rutinosidase for the hydrolysis of rutin into quercetin 3-*O*-glucoside, quercetin, and leucocyanidin (Yang et al., 2012; Riva et al., 2020; Ferreira-Lazarte et al., 2021). Quercetin 3-*O*-glucoside is better absorbed than other forms of quercetin and can suppress the inflammatory response in mice with TNBS-induced colitis *via* the inhibition of the NF-κB and MAPK signaling pathways (Zhang D. et al., 2019). *Bacteroides* sp. 54 metabolizes quercitrin to hydroxyquercitrin and desmethylquercitrin. Quercitrin is also degraded to quercetin by α-L-rhamnosidase and undergoes further ring-cleavage to yield 3,4-dihydroxybenzoic acid by *Bacteroides* sp. 45 (Jiang et al., 2014). β-glucuronidase, which is expressed by *Bacteroidetes* J-37, metabolizes GL to 18β-GA (Kim et al., 1999; Guo et al., 2018). Based on the review of existing studies, natural products are biotransformed under the action of *Bacteroidetes* to produce metabolites with different functional activities. It is important to understand the whole process of natural products occurring in the body to assess the effect on human health.

### Streptococcus

The *Streptococcus* species are Gram-positive, spherical, or ovoid cells, which are usually arranged in chains or pairs and widely exist in human feces and nasopharynx (Lannes-Costa et al., 2021). Meta-transcriptomic analysis indicates that the phosphotransferase system is majority expressed by *Streptococcus*, suggesting that these bacteria are the main utilizers of the available carbohydrates in the small intestinal (Zoetendal et al., 2012). *Streptococcus* LJ-22 expresses β-glucuronidase to metabolize GL to 18β-glycyrrhetinic acid-3-*O*-β-D-glucuronic acid (GAMG; Kim et al., 2000; Park et al., 2004; Guo et al., 2018). GAMG has anti-allergic activity against LPS-induced RAW264.7 cells with IC50 value of 0.28 mM (Park et al., 2004). In addition, tannic acid is degraded by tannase of *Streptococcus gallolyticus* subsp. *Gallolyticus* (SGG) to produce pyrogallol. SGG may contribute to the development of colorectal cancer by eliminating the toxicity of tannic acid to tumor cells (Oehmcke-Hecht et al., 2020). Therefore, further *in vivo* studies are necessary to determine whether the elimination of these tannic acid-degrading microbes can support the effective treatment of colorectal cancer. *S. thermophilus* GIM 1.321 has a high production capacity of β-glucosidase for the degradation of fructus anthocyanins into CHA, CAA, and ferulic acid (Cheng J.R. et al., 2016). The administration of CAA and CHA (10/15 mg/kg/day) can lower blood pressure and exert an anti-oxidant effect (Agunloye et al., 2019). *Streptococcus* strains might be a commensal, pathogenic, and opportunistic pathogen in the gut, and more information is needed about its effect on human health. A better understanding of how *Streptococcus* metabolizes natural products may allow the regulation of the gut microbiome to improve therapeutic efficacy.

### Blautia

*Blautia* species are strictly anaerobic, nonmotile, usually spherical or oval, and widely found in the gut and feces of mammals (Liu X. et al., 2021). There is increasing evidence for the probiotic properties of *Blautia* on the biotransformation of natural products (Tremaroli and Bäckhed, 2012). In the course of flavonoid biotransformation, the reactions catalyzed by *Blautia* include demethylation, *O-*and *C-* deglycosylation, and C-ring cleavage (Braune and Blaut, 2016), which may be catalyzed by the corresponding enzymes, such as *O*-glycosidase and β-glucosidases (Braune et al., 2016). Research indicates that the strain *Blautia* sp. MRG-PMF1 has a hydrolytic ability on aryl methyl ether functional groups by converting 5,7-dimethoxyflavone and 5,7,4-trimethoxyflavone into bioactive chrysin and apigenin, respectively. *Blautia* sp. MRG-PMF1 also possesses deglycosylation activity, and various isoflavones, flavones, and flavones were found to be metabolized into the corresponding aglycones (Kim et al., 2014). Besides, under anaerobic conditions, *Blautia* sp. MRG-PMF1 strain metabolizes icariin further to desmethylicaritin with estrogenic effects (Wu et al., 2016). The strain can also catalyze curcumin to produce demethoxycurcumin with anti-inflammatory and anti-cancer properties (Burapan et al., 2017a; Hatamipour et al., 2019). In addition, *Blautia* sp. AUH-JLD56 is capable of solely biotransforming arctiin or arctigenin into demethylated products with better antioxidant capacity (Liu et al., 2013). Recently, a growing academic interest has been witnessed in the biotransformation and metabolism of herbal plants and functional foods by *Blautia*. Exploring the biotransformation of *Blautia* is of great significance for the development of new enzymes and bioactive metabolites (Meng et al., 2020).

## Key transformation types involved in natural products microbial metabolism

Complex microbial enzymes catalyze the metabolism of natural products in the gut, resulting in lipophilic and low-molecule-weight metabolites conducive to host utilization/excretion (Weersma et al., 2020). Unlike human genetics, the gut microbiome is modifiable in terms of characteristics, making it a potential therapeutic target to optimize therapy. After oral natural products enter the digestive tract, they will first come into contact with a large number of gut microbes and the active enzymes produced by them. Therefore, natural products’ gut biotransformation may occur before the first-pass effect through the liver (Xie et al., 2020). Natural products can be modified/deconjugated by the gut microbiome, and can also be transported to the liver to modify/bind and then excreted into the gut to react with gut microbes to form a series of metabolites (Koppel et al., 2017). The metabolites transformed by the host-microbial co-metabolic system may be functionally novel and not clearly defined. Therefore, the combination of specific strains, specific metabolic pathways, and specific enzymes associated with health/disease is important for the determination of the effect of gut microbes on the host.

### Hydrolysis

**Figure 2:**
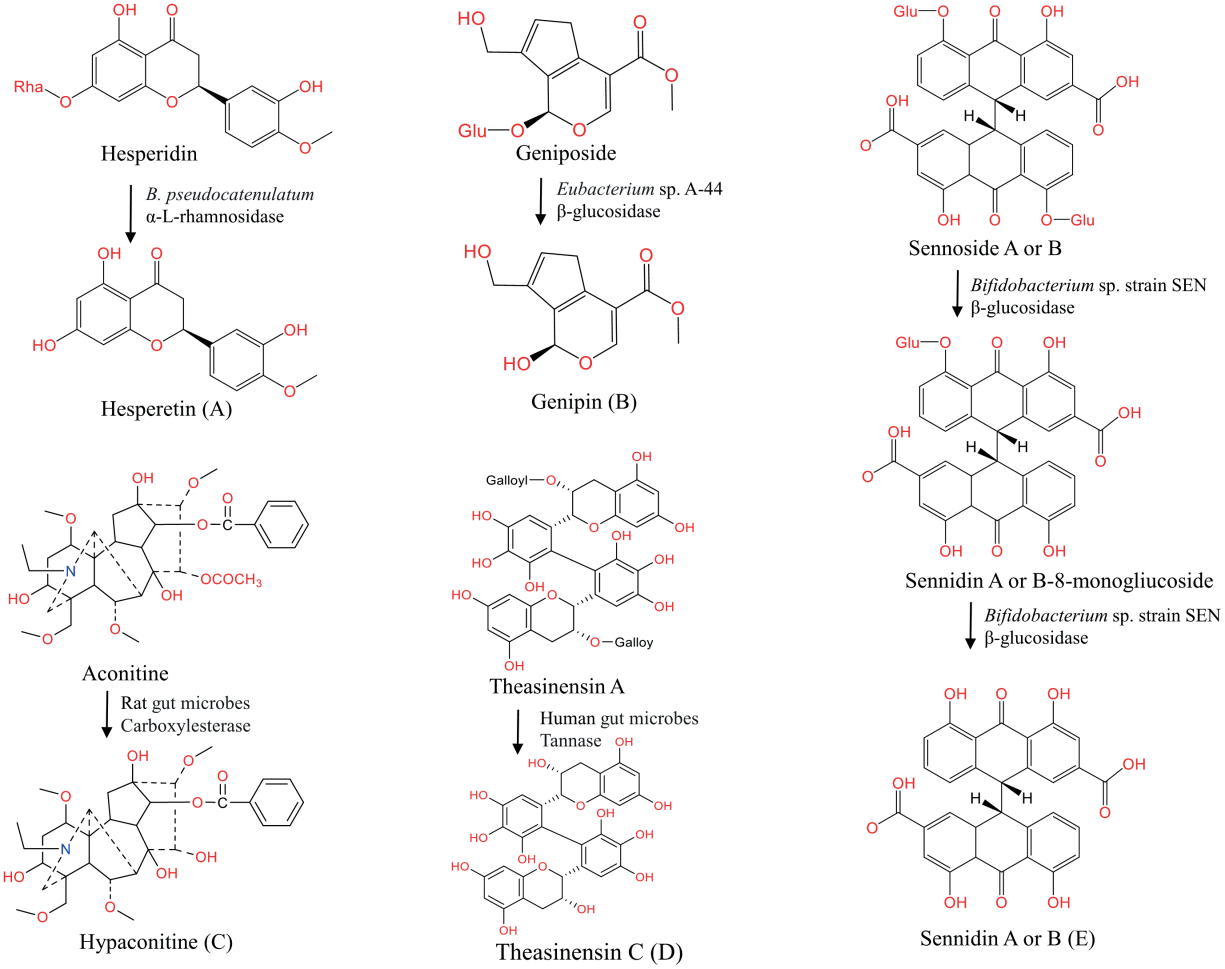
Hydrolysis of natural products under the action of gut microbes. **(A–E)** Hydrolysis of hesperidin (Mas-Capdevila et al., 2020), geniposide (Jiang et al., 2016), aconitine (Zhang et al., 2015), theasinensin A (Liu Z. et al., 2021), sennoside A or B (Matsumoto et al., 2012).

Certain natural products have high molecular weight and low lipid solubility, and they are difficult to be absorbed by the body in the intestine and have low bioavailability (Hostetler et al., 2017). Through gut microbes-mediated hydrolysis, their physical properties are changed, and their biological activity and bioavailability are greatly improved (Wu and Tan, 2019). Slámová et al. indicated that most glycosides have low activity and are considered “natural prodrugs” (Slámová et al., 2018). After interacting with gut microbes, the sugar groups of glycosides are removed, and then, the aglycone portion is absorbed by intestinal cells to exert physiological effects (Wilson and Nicholson, 2017; Murota et al., 2018). The hydrolysis reaction is required for further transformation, and the products (e.g., sugars) participate in promoting the growth and survival of gut microorganisms (Theilmann et al., 2017). Figure 2 shows the hydrolysis reaction of partial natural products under the action of gut microbes.

#### Flavonoids

Flavonoids are natural phenolic compounds found abundantly in fruits and vegetables. Gut microbes may be partly responsible for the efficacy of flavonoids (glycoside forms), which have low bioavailability because of the presence of water-soluble sugar components (Murota et al., 2018; Al-Ishaq et al., 2021). Flavanols with 3-hydroxyflavone base (3-hydroxy-2-phenylchromen-4-one) and planar ring system constitute a significant class of flavonoids. In the study of Du et al., isorhamnetin-3-*O*-neohesperidoside was first deglycosylated to isorhamnetin-3-*O*-glucoside and subsequently to the aglycone isorhamnetin by *Escherichia* sp. 23 (Du et al., 2017). The gut microbes and derived enzymes (lactase phlorizin hydrolase) jointly controlled the metabolism of epimedium koreanum nakai-prenylated flavonoids as determined by *in vitro* assays. In the present study, gut enzymes metabolized flavonoids faster than gut microbes (Zhou et al., 2013). Wu et al. found that α-L-rhamnosidase from *Bacteroides thetaiotaomicron* VPI-5482 could hydrolyze the α-1,2 glycosidic bond of epimedin C to produce icariside (Wu et al., 2018). β-xylosidase Dt-2,286, which is derived from *Dictyoglomus turgidum*, is highly active in hydrolyzing xylose and glucose groups in epimedium B to obtain baohuoside I and sagittatoside B (Tong et al., 2021). Flavanones have a 2,3-dihydro-2-phenylchromen-4-one structure. Hesperidin is converted to its active form hesperetin by α-L-rhamnosidase, which is expressed by *B. pseudocatenulatum* (Mas-Capdevila et al., 2020). Isoflavones are mainly found in legumes. *B. breve* MCC1274 possesses the highest β-glucosidase activity for the conversion of daidzin to daidzein (Yao et al., 2019). The anthocyanin cyanidin 3-glucoside is converted to cyanidin by *E. ramulus* and *Clostridium saccharogumia* (Hanske et al., 2013). Human gut enzymes such as β-glucuronidase play a key role in the hydrolysis of wogonoside to its aglycone form wogonin (Xing et al., 2014). Theasinensin A, a bioactive catechin dimer found in black tea, is degalloylated to yield theasinensin C by human fecal microbiota (Liu Z. et al., 2021). In the present study, we observed the metabolic differences in flavane-3-ols, and the results suggest that steric hindrance may limit the degradation of partial flavane-3-ols C-ring by bacterial enzymes during gut microbial fermentation. Many other flavonoids can also undergo hydrolysis reactions under the action of gut microbes, as shown in Table 1. Notably, considering the structural differences of flavonoids, the degree of degradation of flavonoids by gut microbes varies greatly, thus affecting their bioaccessibility. Further efforts are required to investigate the role of gut metabolism in the bioavailability and absorption of flavonoids and the possible bacteria-flavonoid interaction ctivities.

**Table 1:** Hydrolysis reaction of gut bacteria to natural products.

Classification	Gut microbiota	Enzyme	Substrate	End-product	Changes	Ref.
Flavonoid glycosides	*E. coli* HGU-3; *L. brevis* RO1	β-glucuronidase	Baicalin	Baicalein; oroxylin A	Bioavailability↑ anti-inflammation↑	Yim et al. (2004), Trinh et al. (2010) and Han et al. (2016)
	*E. cellulosolvens* ATCC 43171^T^	β-glucosidase	Luteolin 7-*O*-glucoside; apigenin 7-*O*-glucoside	Luteolin; apigenin	Bioavailability↑	Braune and Blaut (2012) and Braune et al. (2016)
	*E. cellulosolvens* ATCC 43171^T^	NA	Luteolin 6-*C*-glucoside; apigenin 6-*C*-glucoside	Luteolin; apigenin	Bioavailability↑	Braune and Blaut (2012) and Braune et al. (2016)
	Human gut microbes	β-glucuronidase	Wogonoside	Wogonin	Anti-inflammation↑	Xing et al. (2014)
	*Bacteroides* JY-6; *Fusobacterium* K-60	β-glucosidase α-L-rhamnosidase β-rutinosidase	Rutin	Quercetin-3-*O*-glucoside; quercetin; leucocyanidin	Bioavailability↑ anti-oxidant↑	Riva et al. (2020) and Ferreira-Lazarte et al. (2021)
	*Escherichia* sp. 23	β-glucosidase	Isorhamnetin-3-*O*-neohesperidoside	Isorhamnetin-3-*O*-glucoside; isorhamnetin	Bioavailability↑ anti-inflammation↑	Du et al. (2017)
	Rat gut microbes; *B. thetaiotaomicron* VPI-5482	β-glucosidase α-L-rhamnosidase	Epimedin A, B, C	Icariin II; icariin A, B	Anti-osteoporosis↑	Cui et al. (2013), Cui et al. (2014) and Wu et al. (2018)
	*Dictyoglomus turgidum*	β-xylosidase Dt-2,286	Epimedium B	Baohuoside I; sagittatoside B	Anti-osteoporosis↑	Tong et al. (2021)
	*B. animalis* subsp. *lactis* AD011	β-glucosidase	Quercetin 3-*O*-glucoside isorhamnetin 3-*O*-glucoside	Quercetin; isorhamnetin	Anti-tumor↑ anti-inflammatory↑	Youn et al. (2012)
	*Lactobacillus paracasei* A221	β-glucosidase	Kaempferol-3-*O*-sophoroside	Kaempferol	Anti-aging↑	Shimojo et al. (2018)
	*Enterococcus.* sp. 8B, 8-2,9-2	β-glucosidase	Astilbin	Taxifolin	Cardiovascular protection↑ anti-tumor↑ anti-inflammatory↑	Zhao et al. (2014) and Zhao et al. (2021)
	Human gut microbes; *B. pseudocatenulatum*	α-L-rhamnosidase; β-glucosidase	Hesperidin	Hesperetin	Anti-oxidant ↑ anti-inflammatory ↑	Mas-Capdevila et al. (2020)
	Rat gut microbes	β-glucosidase	Calycosin-7-*O*-β-D-glucoside	Calycosin	Neuroprotection↑ anti-oxidant ↑	Ruan et al. (2015)
	*E. ramulus*; *B. breve* MCC1274	β-glucosidase	Daidzin	Daidzein	Neuroprotection↑	Mace et al. (2019) and Yao et al. (2019)
	*Dorea* species PUE	*C*-deglycosylation enzymes (DgpB-C)	3″-*oxo*-puerarin	Daidzein	Bioavailability↑	Nakamura et al. (2020)
	*E. ramulus*; *Clostridium saccharogumia*	β-glucosidase	Cyanidin 3-glucoside	Cyanidin	Bioavailability↑	Hanske et al. (2013)
	Human gut microbes	Tannase	Theasinensins A	Theasinensins C	Bioavailability↑	Liu Z. et al. (2021)
	*Bacillus* sp. KM7-1; *Bacteroides* sp. MANG	C-C glucosyl-cleaving enzyme	Mangiferin	Norathyriol	Anti-cancer↑anti-diabetes↑	Hasanah et al. (2021)
	Strain CG19-1	NA	Mangiferin	Norathyriol		Braune and Blaut (2011)
Terpenoids	*Eubacterium.* sp. A-44	β-glucosidase, carboxylesterases	Geniposide	Genipin; geniposidic acid	Bile secretion↑ anti-hepatitis↑	Akao et al. (1994), Jiang et al. (2016) and Tian et al. (2013)
	*B. fragilis*; *L. brevis*; rat gut microbes	β-glucosidase	Paeoniflorin	PM-I; albiflorin and its aglycone; Deacyl-paeonifloridin	Anti-convulsant↑ Bioavailability↑	He et al. (2007) and Ke et al. (2016)
	Rat gut microbes	Glycoside hydrolases	Asiaticoside	Corresponding aglycones	Bioavailability↑	Weng et al. (2006)
	*Eubacterium* sp. A-44	β-glucosidase; β-D-fucosidase	Saikosaponin B1	Prosaikogenin; saikogenin A	Anti-inflammatory↑ Anti-oxidant↑	Kida et al. (1998)
	*Eubacterium* L-8; *Bacteroidetes* J-37; *Streptococcus* LJ-22	β-glucuronidase	GL	18β-GA; GAMG	Anti-platelet aggregation↑ anti-allergic↑ anti-tumor↑ anti-bacterial↑	Kim et al. (1999), Kim et al. (2000), Park et al. (2004) and Guo et al. (2018)
	*Eubacterium* sp. A-44; *B. breve* ATCC 15700	β-glucosidase	Ginsenoside Rh2	Ginsenoside F_2;_ compound K	Bioavailability↑	Zhong et al. (2016), Zhang R. et al. (2019) and Kim (2018)
	*B. breve*; *B. longum*	Esterases	Albiflorin	Benzoic acid	Anti-depression↑	Zhao et al. (2018) and Peng et al. (2022)
	Human/rat gut microbes	β-glucosidase; α-L-rhamnosidase	Ardipusillosides I	Deglycosylated product	Bioavailability↑	Cao et al. (2015)
	Human gut microbes	NA	Mogroside III	Mogroside II mogrol	Bioavailability↑	Yang et al. (2007)
	*B. adolescentis*; *B. breve*	NA	Pedunculoside	Deglycosylated products	Bioavailability↑	Wu et al. (2019)
	Rat gut microbes	NA	Capilliposide C	Deglycosylated products esterolysis products		Cheng Z. et al. (2016)
Anthraquinones	*Bifidobacterium* sp. strain SEN	β-glucosidase	Sennoside A and B	Sennidin A/B-8-monoglucoside	Purgation↑	Matsumoto et al. (2012)
Alkaloids Phenols	Human gut microbes	CEs	DDAs	MDAs	Toxicity↓	Zhang et al. (2015)
	Rat gut microbes	NA	Scopolamine	Scopine	Anti-tumor↑ anti-inflammatory↑	Dey (2019)
	*L. plantarum*	Tannase	Gallic tannins	Gallic acid	Anti-oxidant↑ anti-inflammatory↑	Jiménez et al. (2014)
	*Akkermansia muciniphila*	Tannase	Ellagitannins	Ellagic acid	Neuroprotection↑	Luca et al. (2020)
	Rat gut microbes	β-glucosidase	Amygdalin	Mandelonitrile; prunasin; phenylacetonitrile; hydrogen cyanide	Toxicity↑	Kim et al. (2008) and Qin et al. (2021)
	*B. animalis*	Feruloyl esterase	CHA	CAA	Anti-oxidant↑	Raimondi et al. (2015)
	*L. plantarum*; *L. johnsonii*; *L. acidophilus*	Feruloyl esterases	CAA; p-coumaric acids	ferulic acid	Anti-oxidant↑	Fritsch et al. (2017)
	*E. coli* Nu; *E. coli* MC; *E. coli* WC-1	Cinnamyl esterase	Conjugated hydroxycinnamates	Free hydroxycinnamates	Anti-oxidant↑ anti-cancer↑	Couteau et al. (2001)
Steroids	Human gut microbes	NA	Pulsatilla saponin D	Corresponding deglycosylation products	Bioavailability↑	Yan et al. (2018)
Other	Mouse gut microbes	β-glucosidase	Cycasin	Diazomethane	Toxicity↑	Goldin (1990)

#### Terpenoids

Terpenoids are the largest class of natural products with anti-cancer, anti-inflammatory, and neuroprotective effects (Agatonovic-Kustrin et al., 2020; El-Baba et al., 2021). Part of terpenoids can also be hydrolyzed by gut microbes. Geniposide produces genipin with the action of β-glucosidase expressed by *Eubacterium* sp. A-44 (Akao et al., 1994; Jiang et al., 2016). Paeoniflorin is transformed into PM-I under the action of β-glucosidase, which is expressed by *L. brevis* and *B. fragilis* (Abdel-Hafez et al., 1999; He et al., 2007). By incubating with rat anaerobic gut microbiota, paeoniflorin is also deglucosed and dephenyled into albiflorin and acyl albiflorin with a small molecular weight (Ke et al., 2016). Peng et al. demonstrated that several *Bifidobacteria* species with esterase can hydrolyze albiflorin to benzoic acid *in vitro* (Peng et al., 2022). *In vitro* study shows that asiaticoside is gradually deglycosylated by glycoside bond hydrolase and produces corresponding aglycones (Weng et al., 2006). Saikosaponin B1 is gradually hydrolyzed to prosaikogenin and saikogenin A under the action of β-glucosidase and β-*D*-focusidase, which are expressed by *Eubacterium* sp. A-44 (Kida et al., 1998). Except for the compounds mentioned above, terpenoids ginsenoside Rh2 (Guo et al., 2019), ardipusillosides I (Cao et al., 2015), mogroside III (Yang et al., 2007), and pedunculoside (Wu et al., 2019) can also undergo hydrolysis reactions under the action of gut microbes (Table 1). Therefore, gut microbes play an important role in terpenoid metabolism, and the effects of their metabolites on gut microbiome and human health need to be further studied.

#### Other compounds

Ellagitannins, which have a very low bioavailability perform a pharmacological role only when it is hydrolyzed into derivatives such as ellagic acid and uroliths under the action of tannase from *Gordonibacter urolithinfaciens*, *Gordonibacter pamelaeae*, and *Ellagibacter isourolithinifaciens* (Beltrán et al., 2018; García-Villalba et al., 2020; Tang et al., 2021). The anthraquinone glycosides extracted from rhubarb are hydrolyzed into anthraquinone aglycones by gut microbes (Li Q. et al., 2020). Sennoside A, a major component of rhubarb extract, is metabolized into rhein anthrone by β-glucosidase of *Bifidobacterium* sp. strain SEN (Matsumoto et al., 2012; Kon et al., 2014). Under the action of carboxylesterase (CEs), which are expressed by gut microbes, diester diterpenoid alkaloids (DDAs, such as aconitine) hydrolyze the ester bonds of C-8 and C-14 to produce monoester diterpene alkaloids (MDAs, such as hypaconitine), which are less toxic (Zhang et al., 2015). *Pulsatilla Chinensis* is commonly used in Asia, and its major saponin anemoside B4 can be degraded by gut microbes to produce deglycosylation products (Wan et al., 2017). Table 1 shows that the alkaloids scopolamine (Wu et al., 2019), steroid compound pulsatilla saponin D (Yan et al., 2018), and cycasin (Goldin, 1990) undergo hydrolysis reactions under the action of gut microbes. The hydrolysis reaction is an important step in the metabolism of natural products by gut microbes and is required for the expression of biological activity and further biotransformation. The specific microorganisms and enzymes involved in this reaction should be focused on to fully understand the ultimate fate of natural products and their impact on human health and provide a basis for personalized treatment.

### Methylation and demethylation

**Figure 3:**
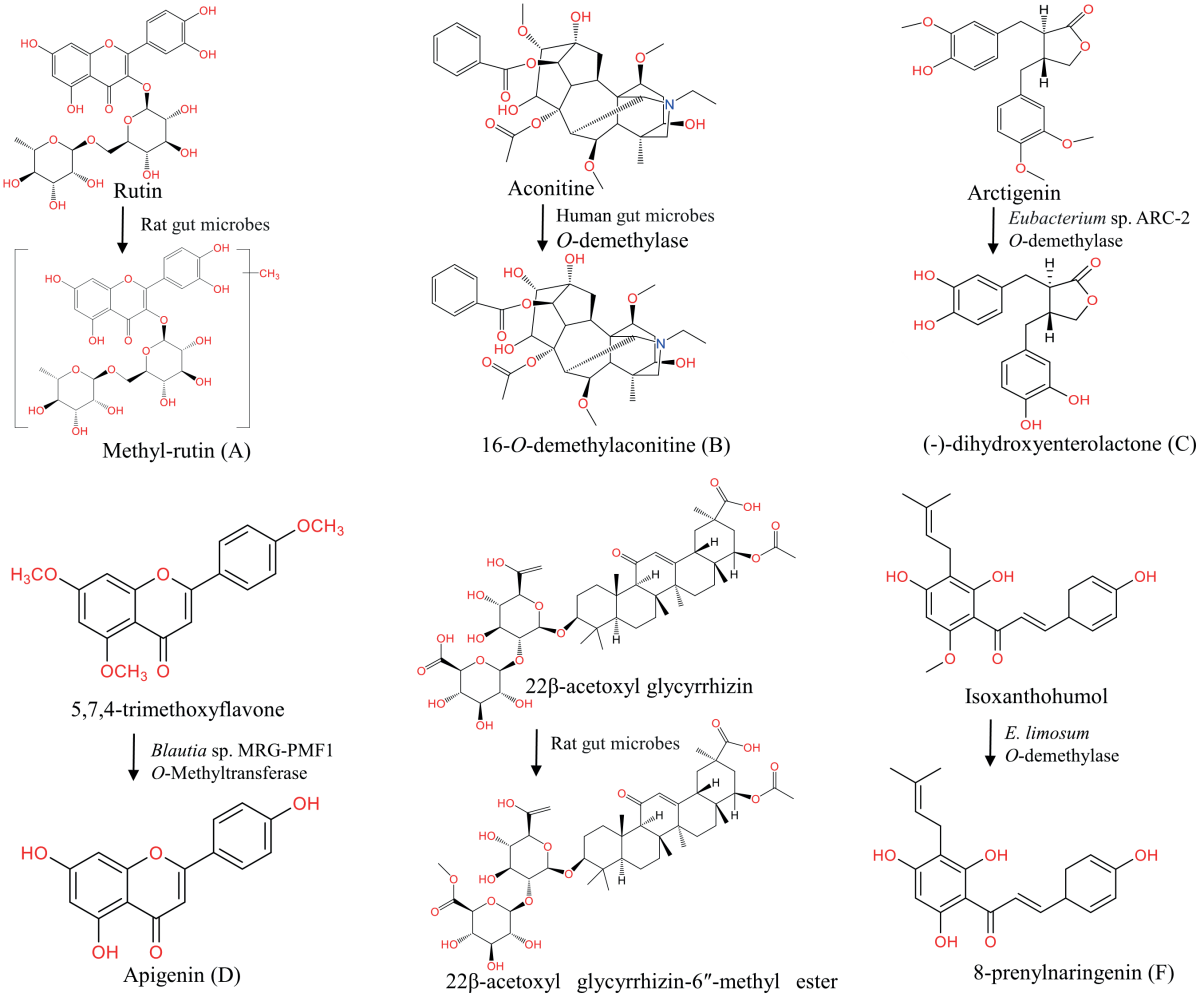
Methylation and demethylation of natural products under the action of gut microbes. **(A)** Methylation of rutin (Wu et al., 2017); **(B–F)** Demethylation of aconitine (Zhang et al., 2017), arctigenin (Jin et al., 2013), 5,7,4-trimethoxyflavone (Kim et al., 2014), 22β-acetoxyl glycyrrhizin (Wang Q. et al., 2015), isoxanthohumol (Paraiso et al., 2019).

Gut microbes can express transferases and move functional groups between the two substrates through nucleophilic substitution reactions (Koppel et al., 2017). The addition of methyl to exogenous substances by gut microbes requires chemically activated co-substrates, such as acetyl coenzyme A, adenosine triphosphate, or S-adenosylmethionine, while demethylation requires cofactors that can undergo nucleophilic catalysis, such as COB (I) alamin, and tetrahydrofolate (Kumano et al., 2016). Methylation modification can optimize the physiological activity of natural products, and demethylation can release polar groups for further binding and excretion from the body, and provide a carbon source for the growth of gut microbes (Ticak et al., 2014). Figure 3 shows the methylation and demethylation of natural products under the action of gut microbes.

#### Flavonoids

The methylation modification can be carried out at the C-2, C-3, C-4, C-5, C-6, C-7, and C-8 positions in the structure of flavonoids, and the bioavailability of methylated flavonoids is greatly improved (Wen and Walle, 2006). Bernini et al. found that *O*-methylated flavonoids have remarkable anti-cancer activity and resistance to hepatic metabolism (Bernini et al., 2011; Choi, 2019). After oral administration of rutin in rats, many methylated metabolites, such as methylrutin, methylisoquercetin, and methylquercetin sulfate, are detected in fecal samples (Yang et al., 2012; Wu et al., 2017; Riva et al., 2020). Methoxylated isoflavonoids formononetin and biochanin A undergo demethylation to produce daidzein and genistein under the action of *E. limosum* ATCC 8486 (Hur and Rafii, 2000). Isoxanthohumol yields demethylation products 8-prenylnaringenin by *E. limosum* (Paraiso et al., 2019). Hesperidin, hesperetin (Pereira-Caro et al., 2018; Jiao et al., 2020), 5,7-dimethoxyflavone, xanthohumol (Paraiso et al., 2019), and 5,7,4′-trimethoxyflavone (Kim et al., 2014) can also undergo demethylation reactions under the action of gut microbes (Table 2).

**Table 2:** Methylation and demethylation reaction of gut microbes to natural products.

Classification	Gut microbiota	Enzyme	Substrate	End-product	Changes	Ref.
Flavonoids	Rat gut microbes	Methyltransferase	Rutin	Methylrutin; Methyl-isoquercetin; methylquercetin sulfate	Bioavailability↑	Wu et al. (2017)
	Mice gut microbes	NA	Myricetin	Mono- and di-methylated myricetin	Toxicity↓	Zhang et al. (2020)
	Rat gut microbes	NA	Hesperidin; hesperetin	Demethylated products	Bioavailability↑	Pereira-Caro et al. (2018) and Jiao et al. (2020)
	*E. limosum*	*O*-demethylase	Formononetin; biochanin A	Daidzein; genistein	Estrogen effect↑	Hur and Rafii (2000)
	*Blautia* sp. MRG-PMF1	Methyltransferase	5,7-dimethoxyflavone; 5,7,4′-trimethoxyflavone	Chrysin; apigenin	Anti-oxidant↑ anti-inflammatory↑ anti-cancer↑	Kim et al. (2014)
	*Blautia* sp. MRG-PMF1	Methyltransferase	Icariin	Desmethylicaritin	Estrogenic effects↑	Wu et al. (2016)
	*E. limosum*	*O*-demethylase	Isoxanthohumol	8-prenylnaringenin	Anti-androgen↑ anti-osteoporosis↑	Paraiso et al. (2019)
Alkaloids	Human gut microbes	Methyltransferase	Quassic ketone	Quassic alkali butyl		Chen F.Z. et al. (2021)
	Rat gut microbes	Methyltransferase	Palmatine	Columbamine; Jatrorrhizine; demethyleneberberine	Bioavailability↑	He et al. (2017) and Liao et al. (2021)
	Human gut microbes	*O*-demethylase	Aconitine	16-*O*-demethylaconitine	Toxicity↓	Zhao et al. (2008) and Zhang et al. (2017)
Lignans	*Eubacterium* sp. ARC-2; *Blautia* sp. AUH-JLD56	*O*-demethylase	Arctiin; arctigenin	DHENL; 3′-DMAG	Anti-oxidant↑ estrogen effect↑	Jin et al. (2007), Jin et al. (2013), Liu et al. (2013) and Seyed Hameed et al. (2020)
	*Blautia producta* DSM3507; *Gordonibacter* strains 3C and 28C; *Lactonifactor longoviformis* DSM17459^T^	Guaiacol lignan methyltransferase; catechol lignan dehydroxylase; enterodiol lactonizing enzyme	Secoisolariciresinol	Enterolactone; enterodiol	Estrogen effect↑	Bess et al. (2020) and Tse et al. (2022)
	Human gut microbes	SesA	Sesamin	Enterolactone; enterodiol	Estrogen effect↑	Peñalvo et al. (2005)
	Rat gut microbes	*O*-demethylase	Matairesinol	2,3-bis(3,4-dihydroxybenzyl) butyrolactone; enterolactone	Anti-inflammatory↑ estrogen effect↑	Clavel et al. (2006), Yamawaki et al. (2011) and Michalak et al. (2018)
	Human/rat gut microbes	*O*-demethylase	Phillygenin	Enterolactone	Anti-inflammatory↑ estrogen effect↑	Michalak et al. (2018)
	*E. limosum* ZL-II; human gut microbes	*O*-demethylase	Silybin A and B	Demethylsilybin A; demethylsilybin B	Anti-Alzheimer’s disease↑	Zhang et al. (2014) and Valentová et al. (2020)
Diketones	*Blautia* sp. MRG-PMF1	Co *O*-Methyltransferase	Curcumin	DMC; bDMC	Anti-tumor↑ anti-inflammatory↑	Burapan et al. (2017a,b)
Phenol**s**	Rat gut microbes	NA	Danshensu	3-(3-*O*-methyl-4-hydroxyphenyl)-2-hydroxypropanoic acid	Bioavailability↑	Gu et al. (2014)
	Rat gut microbes	*O*-demethylase	Dihydro-isoferulic acid	Dihydrocaffeic acid	Anti-oxidant↑ anti-apoptosis↑	Kay et al. (2017)
Terpenoids	Rat gut microbes	NA	22β-acetoxyl glycyrrhizin	22β-acetoxyl glycyrrhizin-6″-methyl ester	Bioavailability↑	Wang Q. et al. (2015)
	*Eubacterium* sp. A-44	NA	Genipin	Geniposidic acid	Anti-oxidant↑	Akao et al. (1994)
Stilbenoids	Human gut microbes	*O*-demethylase	Thunalbene	Isoresveratrol	Anti-oxidant↑	Jarosova et al. (2019)
Steroids	Human gut microbes	NA	Pulsatilla saponin B3	Corresponding Deglycosylation products	Bioavailability↑	Liu et al. (2015)

#### Alkaloids

Alkaloids are nitrogen-containing compounds, which are biosynthesized by both marine and terrestrial organisms, and they have anti-cancer (Tse et al., 2022) and anti-viral activity (Abookleesh et al., 2022). Under the action of enzymes expressed by gut microbes, quassic ketone, the main alkaloid component in bitter wood, is methylated into quassic alkali butyl (Fan et al., 2013; Chen et al., 2021). Isoquinoline alkaloid palmatine yields demethylation products such as columbamine, jatrorrhizine, demethyleneberberine, and demethyleneberberine *via in vitro* anaerobic incubation (He et al., 2017; Liao et al., 2021). The demethylation of aconitine by gut microbes is demonstrated by ion trap electrospray ionization tandem mass spectrometry, and 16-*O*-demethylaconitine is produced (Zhao et al., 2008; Zhang et al., 2017).

#### Lignans

Dietary lignans are phytoestrogens that are mostly found in seeds, nuts, legumes, and vegetables. Arctiin can be demethylated to (−)-dihydroxyenterolactone (DHENL) and other products by *Eubacterium* sp. ARC-2 strain (Jin et al., 2007, 2013; Seyed Hameed et al., 2020). Liu et al. isolated a bacterium named *Blautia* sp. AUH-JLD56 from human fecal bacteria, and this species could efficiently transform arctiin or arctigenin into a demethylation metabolite 3′-desmethylarctigenin (3′-DMAG; Liu et al., 2013). Secoisolariciresinol, which is one of the most common lignans found in flaxseed, can be demethylated in the presence of *Blautia producta*, *Gordonibacter* and *Lactonifactor longoviformis* to form enterolactone and enterodiol (Bess et al., 2020; Tse et al., 2022). Sesamin is metabolized into mammalian lignan enterolactone and enterodiol through methylation, demethylation, and other reactions by gut microbes (Peñalvo et al., 2005). Matairesinol and phillygenin can also be demethylated to produce enterolactone (Clavel et al., 2006; Yamawaki et al., 2011; Michalak et al., 2018). Silybin A and B are demethylated into demethylsilybin A and demethylsilybin B by human fecal microbiota (Zhang et al., 2014; Valentová et al., 2020).

#### Other compounds

Polyphenol compound curcumin is demethylated by *Blautia* sp. MRG-PMF1 to produce metabolites demethoxycurcumin (DMC) and bis-demethoxycurcumin (bDMC; Burapan et al., 2017a,b). The demethylated products of dihydro-isoferulic acid, such as dihydrocaffeic acid, are also obtained in fecal metabolites (Kay et al., 2017). Wang et al. found that the methylation reaction occurs at the internal and external glucuronic acid residues of the licorice saponins 22β-acetoxyl glycyrrhizin sugar chain, yielding 22β-acetoxyl glycyrrhizin-6″-methyl ester (Wang et al., 2015). Compounds such as polyphenols danshensu (Gu et al., 2014), terpenoids genipin (Akao et al., 1994), stilbenoids thunalbene (Jarosova et al., 2019), and steroids pulsatilla saponin B3 (Liu et al., 2015) undergo methylation and demethylation under the action of gut microbes, as shown in Table 2. Methylation and demethylation reactions are important pathways of gut microbial metabolism, and have been confirmed in many studies. However, the genes/enzymes that mediate this reaction have not been fully characterized.

### Redox reaction

**Figure 4:**
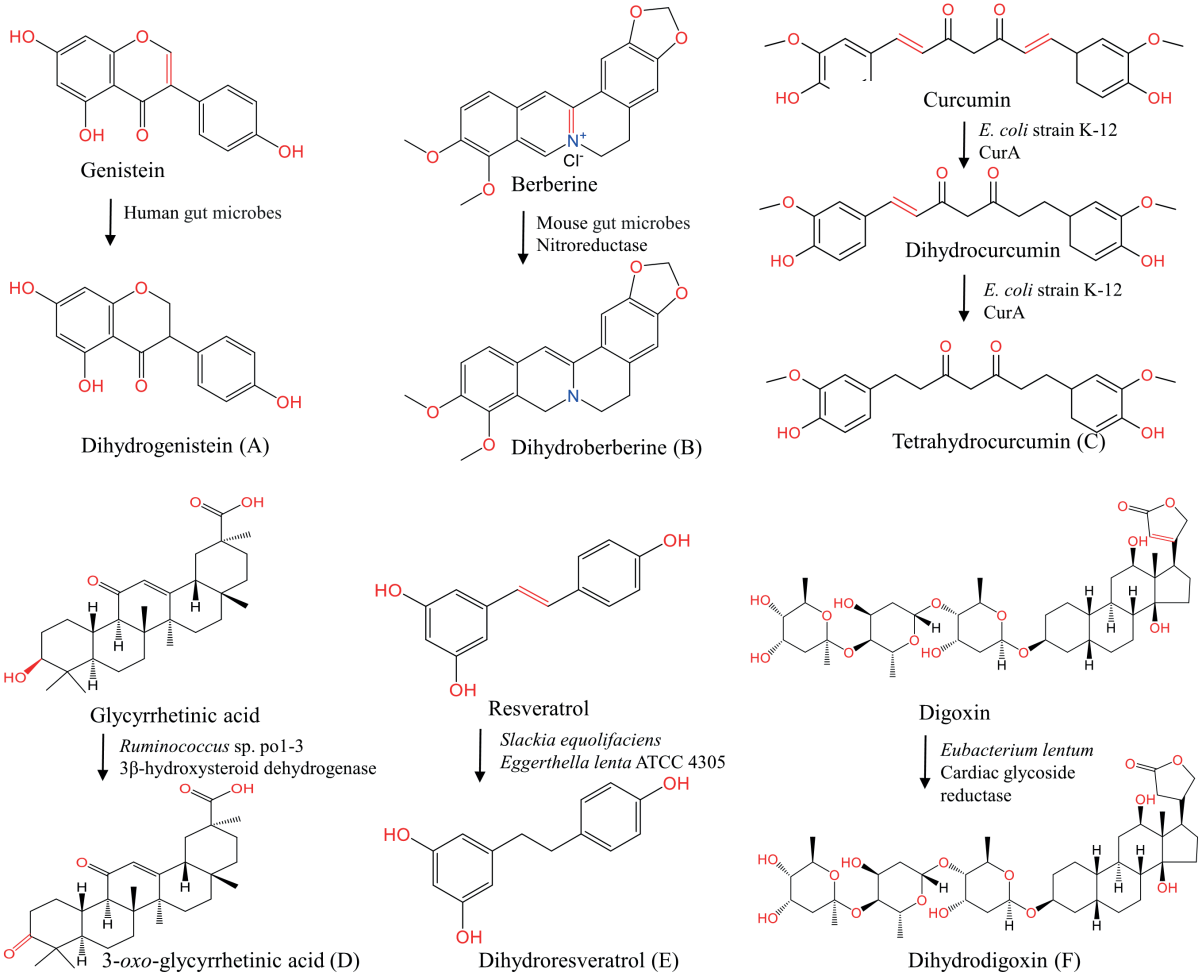
Oxidation and reduction of natural products under the action of gut microbes. **(A–C, E, F)** Reduction of genistein (Mace et al., 2019), berberine (Feng et al., 2015), curcumin (Hassaninasab et al., 2011), resveratrol (Pallauf et al., 2019), digoxin (Kumar et al., 2018); **(D)** glycyrrhetinic acid.

Gut microbes can express many oxidoreductases and transform natural compounds by adjusting various functional groups, such as olefins, carboxylic acid derivatives, nitro, *N*-oxides, and a, b-unsaturated carboxylic acid derivatives, which influence the activity of natural products *in vivo* (Lavrijsen et al., 1995; Haiser et al., 2013; Abookleesh et al., 2022). Various cofactors such as NADH, NADPH, flavin, Fe/S cluster, heme, and molybdenum cofactor are involved in the mediation of the transfer of electron or hydride equivalent (H^+^, 2e^−^) to the substrate (Vanoni, 2021; Lubner et al., 2022). Figure 4 shows the oxidation and reduction reactions of natural products under the action of gut microbes.

#### Flavonoids

Daidzein is reduced to dihydrodaidzein and further tetrahydrodaidzein under the action of *Clostridium* sp. strain HGH6 and *Lactobacillus.* sp. Niu-O16 (Zhao et al., 2011; Heng et al., 2019). The reduced product dihydrogenistein is produced by genistein under the action of human fecal bacteria (Mace et al., 2019). By using UPLC-ESI-Q-TOF-MS/MS analysis, compounds such as the deoxidized metabolites kaempferol and the C2-C3 double bond hydrogenation reduction product taxifolin were identified in the culture solution of rat gut fluid by incubation with quercetin under anaerobic conditions (Qin et al., 2017). Yang et al. discovered a flavone reductase from *Flavonifractor plautii* ATCC 49531, and this enzyme specifically catalyzes the hydrogenation of the C2-C3 double bound of flavones/flavanols C-ring and acts during the initial step of the entire biodegradation pathway of flavonoid (Goris et al., 2021; Yang et al., 2021). *O*-desmethylxanthohumol, a chalcone compound, is reduced to *O*-desmethyl-α, β-dihydroxanthohumol by *E. ramulus* (Paraiso et al., 2019).

#### Alkaloids

Nitroreductase, which is produced by gut microbes, catalyzes ether and coordination bond reactions in alkaloids. Berberine (BBR), as the main component of *Coptis Chinensis*, can be reduced to dihydroberberine (dhBBR) by nitroreductase expressed by gut microbes, and this reduction product has high polarity. dhBBR could be absorbed in the intestine and then oxidized into the prototype BBR into the blood. The absorption rate of dhBBR in the intestine is five times that of BBR (Feng et al., 2015). Li et al. found that the gut microbes could transform BBR into oxyberberine *via* oxidation (Li et al., 2020). Oxyberberine, a novel metabolite of BBR, may be a promising bioactive agent worthy to be explored. Coptisine is a natural protoberberine alkaloid with the same maternal structure as BBR. After oral administration of coptisine, the C-O bond is opened and cracked, followed by a reduction reaction to produce hydrogenated BBR (Cui et al., 2018). Avenanthramide-C is reduced by mice and the human gut microbiota into dihydroavenanthramide-C (Wang et al., 2015).

#### Phenylpropanoids

Caffeic acid (CAA), as the main dietary polyphenol in food and beverage, can easily enter the colon and react with gut microbiota after esterification. CAA is transformed to 3-hydroxyphenylpropionic acid through C4 double bond reduction and dehydroxylation, and then rapidly converted to 3-phenyl propionic acid *via* the β-oxidation of gut microbes *in vitro* (Gonthier et al., 2006). CAA can also be dehydroxylated to m-coumaric acid or hydrogenated to dihydrocaffeic acid (García-Villalba et al., 2020). Danshensu, the major monomer phenolic acid of *Salvia Miltiorrhiza*, undergo dehydrogenation and deoxygenation by gut microbiota to produce 3-phenyl-2-hydroxy propionic acid, 3-(3,4-dihydroxy phenyl) 2-acrylic acid (caffeic acid), and 3-(3,4-dihydroxy phenyl)-propionate (Gu et al., 2014).

#### Other compounds

Glycyrrhetic acid generates 3-*oxo*-glycyrrhetinic acid by 3β-hydroxysteroid dehydrogenase of *Ruminococcus* sp. po1-3 in the cecum. Sennosides, a class of natural anthraquinone derivative and dimeric glycosides, are first hydrolyzed by β-glucosidase to produce sennoside-8-*O*-monoglycoside, and then reduced to rhubaranthrone with purgative effect by *Streptococcus in vivo* (Hattori et al., 1988). Stilbenoids resveratrol is reduced to dihydroresveratrol by *Slackia equolifaciens* and *Eggerthella lenta* ATCC 4305 (Bode et al., 2013; Pallauf et al., 2019) Moreover, diketones curcumin (Hassaninasab et al., 2011; Tan et al., 2014), steroid compounds digoxin (Kumar et al., 2018) and other compounds aristolochic acid (Feng et al., 2019) can also be reduced in the presence of gut microbes (Table 3). Gut microbial flavone reductase and nitroreductase have special catalytic selectivity, filling key gaps in gut microbial transformation pathways. However, the specific genes and enzymes that mediate gut microbial reduction have not been fully determined.

**Table 3:** Reduction and oxidation reaction of gut microbes to natural products.

Classification	Gut microbiota	Enterobacterial metabolic enzyme	Substrate	End-product	Changes	Ref.
Flavonoids	*Clostridium* sp. strain HGH6; *Lactobacillus* sp. Niu-O16	Dihydrodaidzein reductase; tetrahydrodaidzein reductase	Daidzein	Dihydrodaidzein; tetrahydrodaidzein	Anti-osteoporosis↑	Wang et al. (2007) and Heng et al. (2019)
	*Aeroto* Niu-O16	NA	Genistein	Dihydrogenistein	Bioavailability↑	Mace et al. (2019)
	Rat gut microbes *E. ramulus*	Flavone reductase	Quercetin	Kaempferol; taxifolin	Bioavailability↑	Qin et al. (2017)
	*E. ramulus*	Flavanone-/flavanonol-cleaving reductase	Xanthohumol; *O*-desmethylxanthohumol	α, β-dihydroxanthohumol; *O*-desmethyl-α, β-dihydroxanthohumol	Anti-bacterial↑	Paraiso et al. (2019)
Alkaloids	Mouse gut microbes	Nitroreductase	BBR; coptisine	dhBBR; hydrogenated berberine	Bioavailability↑ anti-inflammatory↑	Feng et al. (2015) and Cui et al. (2018)
	Mouse gut microbes	NA	BBR	Oxyberberine	Anti-fungal↑	Li C. et al. (2020)
	Mouse/human gut microbes	NA	Avenanthramide-C	Dihydroavenanthramide-C	Anti-inflammation↑ anti-atherogenesis↑	Wang P. et al. (2015)
Phenolic acids	Human gut microbes	NA	CAA	Dihydrocaffeic acid	Bioavailability↑	Gonthier et al. (2006) and García-Villalba et al. (2020)
	Rat gut microbes	NA	Isoferulic acid	Dihydrocaffeic acid	Anti-oxidant↑ anti-apoptosis↑	Kay et al. (2017)
	Rat gut microbes	NA	Dansensu	3-phenyl-2-hydroxy propionic acid; 3-(3,4-dihydroxy phenyl) 2-acrylic acid; 3-(3,4-dihydroxy phenyl)-propionate	Bioavailability↑	Gu et al. (2014)
	*Gordonibacter urolithinfaciens*	Catechol-dehydroxylase	Chlorogenic acid; rosmarinic acid	Dihydro-chlorogenic acid; dihydro-rosmarinic acid	Bioavailability↑	García-Villalba et al. (2020)
Terpenoids	*Ruminococcus* sp. po1-3	3β-hydroxysteroid dehydrogenase	Glycyrrhetinic acid	3-*oxo*-glycyrrhetinic acid	Anti-inflammatory↑	
Anthraquinone	Human gut microbes; *Streptococcus* spp.	NA	Sennoside-8-*O*-monoglycoside	Rhubaranthrone	Purgation↑	Hattori et al. (1988) and Matsumoto et al. (2012)
Stilbenes	*Slackia equolifaciens*; *Eggerthella lenta* ATCC 4305	NA	Resveratrol	Dihydroresveratrol	Anti-oxidant↑	Jung et al. (2009), Bode et al. (2013) and Pallauf et al. (2019)
Diketones	*E. coli* strain K-12; *E. fergusonii* ATCC 35469; *E. coli strains* ATCC 8739 and DH10B	CurA	Curcumin	DHC; THC	Anti-oxidant↑ lipid-lowering↑	Hassaninasab et al. (2011) and Tan et al. (2014)
Steroids	*Eubacterium lenta*	Cardiac glycoside reductase	Digoxin	Dihydrodigoxin	Bioavailability↓	Kumar et al. (2018)
Other classes	Human gut microbes	NA	Aristolochic acid	Aristololactams	Anti-cancer↑	Feng et al. (2019)

### Other reactions

As shown in Table 4, natural products are also transformed by gut microbes through ring fission, sulfuration, aromatization, and other reactions. Gentiopicroside, a natural iridoid glycoside, can be hydrolyzed to gentianaldehyde by gut microbial β-glucosidase, and then to nitrogen-containing compounds *via N*-heterocyclic reaction (el-Sedawy et al., 1989). The partial ring-opening of genipin acetone alcohol results in the formation of dialdehyde by gut microbes (Kang et al., 2012). Quinic acid can be aromatized to hippuric acid in the presence of gut microbes (Pero and Lund, 2011). Maren et al. incubated kaempferol-*O*-glycosides and apigenin-*C*-glycosides with human fecal samples to generate 3-(4-hydroxyphenyl) propionic acid, 3-phenyl propionic acid, and phenylacetic acid through deglycosylation, ring fission and other reactions (Vollmer et al., 2018). The main hydrolytic and ring-cleaved metabolites, namely, benzoic acid, 2-(3,4-dihydroxy phenyl) acetic acid, and 5-(3,4-dihydroxy phenyl)-γ-valerolactone were obtained *via in vitro* fermentation of flavan-3-ols procyanidin B_2_ and A_2_ with human gut microbes (Stoupi et al., 2010; Ou et al., 2014; Le Bourvellec et al., 2019). Sulfated and hydrogen-reduced metabolites have been detected in the fecal samples of rats after oral administration of luteolin (Li et al., 2017; Káňová et al., 2020). The conversion of daidzein to equol, which is facilitated by gut microbes is another interesting example (Li et al., 2000; Hur et al., 2002; Mayo et al., 2019). *Eggerthella lenta* and *Flavonifractor plautii* reductively cleaved the heterocyclic C-ring of both (−)-epicatechin and (+)-catechin giving rise to 1-(3,4-dihydroxyphenyl)-3-(2,4,6-trihydroxyphenyl) propan-2-ol, δ-(31,41-dihydroxyphenyl)-γ-valerolactone, and δ-(31,41-dihydroxyphenyl)-γ-valeric acid (Ozdal et al., 2016). Tea polyphenols are metabolized by gut microbiota (Liu et al., 2018). Tea polyphenols first undergo structural modifications such as methylation and sulfation in the small intestine and then enter the colon to be cleaved into small phenolic acids, which is conducive to absorption (Cheng et al., 2018). SesA, a sesamin-metabolizing enzyme from *Sinomonas* sp. no. 22, catalyzes the methylene group transfer from sesamin or sesamin monocatechol to tetrahydrofolate with ring cleavage, yielding sesamin mono- or di-catechol and 5,10-methylenetetrahydrofolate (Kumano et al., 2016). The terpenoids astragaloside A (He et al., 2019), flavonoids quercitrin (Jiang et al., 2014) and myricetin (Zhang et al., 2019), the phenol anthocyanidin (Aura et al., 2005), the alkaloid strychnine *N*-oxide (el-Mekkawy et al., 1993) and the aliphatic myristic acid (Du et al., 2014) can all undergo biotransformation reactions to generate active metabolites under the action of gut microbes. These studies demonstrate the enormous metabolic potential of various gut microbiomes. The gut microbial metabolism of natural products and their role in host health should be the focus of future research.

**Table 4:** Other reactions of gut microbes to natural products.

Classification	Gut microbiota	Biotransformation	Enterobacterial metabolic enzyme	Substrate	End-product	Changes	Ref.
Terpenoids	Human gut microbes	Cyclization	β-glucosidase	Gentiopicroside	Gentisaldehyde; nitrogen-containing compounds	Anti-inflammatory↑	el-Sedawy et al. (1989)
	Human gut microbes	Cyclization	NA	Geniposide	Nitrogen-containing compounds	Bioavailability↑	Kawata et al. (1991)
	Human gut microbes	Deglycosylation; deacetylation; dehydrogenation	NA	Astragaloside A	Cycloastragenol	Bioavailability↑	He et al. (2019)
Phenolic acids	Rat gut microbes	Aromatization	NA	Quinic acid	Hippuric acid	Anti-cancer↑ anti-bacterial↑ anti-viral↑	Pero and Lund (2011)
	*Egerthella lenta*	Dehydroxylation	Catechol dehydroxylases	Dihydrocaffeic acid	3-(3-hydroxyphenyl) propionic acid	Bioavailability↑	Maini Rekdal et al. (2020)
	Human gut microbes	Ring cleavage; sulfation methylation	NA	Tea polyphenols	Phenolic acids	Bioavailability↑	Cheng et al. (2018)
	*L. plantarum* WCFS1	Ring fission; hydrolysis	Tannase; gallate decarboxylase	Gallotannins	Gallic acid; pyrogallol	Anti-oxidant↑ anti-inflammatory↑	Reverón et al. (2015) and Fang et al. (2019)
	*SGG*	Ring fission; hydrolysis	Tannase; gallate decarboxylase	Gallotannins	Gallic acid; pyrogallol	Anti-cancer↓	Oehmcke-Hecht et al. (2020)
	*Gordonibacter urolithinfaciens*; *Goronibacter pamelaeae*; *Ellagibacter isourolithinifacens*	Decarboxylation; lactone-ring cleavage; dehydroxylation	NA	Ellagic acid	Urolithins	Anti-cancer↑ anti-oxidant↑ anti-inflammatory↑	Beltrán et al. (2018), García-Villalba et al. (2020) and Tang et al. (2021)
Flavonoids	Rat gut microbes	Sulfation	Aryl sulfotransferase	Luteolin	Luteolin-3′-*O*-sulfate; luteoli*n-*4′-*O*-sulfate		Li et al. (2017) and Káňová et al. (2020)	
	*Clostridium* sp. strain HGH136	C-ring fission	2-dehydro-*O*-demethylangolensin	Daidzein	*O*-desmethylangolensin	Anti-cancer↑	Hur et al. (2002)	
	*Eggerthella* sp. strain YY7918; *B. breve* ATCC 15700 T; *B. longum* BB536; *L. paracasei* CS2	Ring-fission	Dihydrodaidzein racemase	Dihydrodaidzein	S-equol	Estroge effect↑	Yokoyama and Suzuki (2008) and Mayo et al. (2019)	
	*E. ramulus*	Ring-fission; reduction	Chalcone isomerase; flavanone-/flavanonol-cleaving reductase	Naringenin; eriodictyol	Naringenin chalcone; phloretin; 3-hydroxyphloretin	Bioavailability↑ anti-inflammatory↑	Gall et al. (2014) and Braune et al. (2019)	
	*E. ramulus* strain wK1	Ring-fission	Phloretin hydrolase	Phloretin	3-(4-hydroxyphenyl)-propionic acid; phloroglucinol	Bioavailability↑	Schoefer et al. (2004) and Braune et al. (2019)	
	*Bacteroides* sp. 45; *B. fragilis;* *E. ramulus*	Ring-fission	Chalcone isomerase; phloretin hydrolase	Quercetin; luteolin	4-hydroxybenzoic acid 3,4-dihydroxyphenylacetic acid; 3,4-dihydroxybenzoic; 3-(3-hydroxyphenyl) propionic acid	Anti-platelet aggregation↑anti-tumor↑	Jiang et al. (2014) and Braune et al. (2001)	
	Rat gut microbes	Ring-fission sulfation	Chalcone isomerase; phloretin hydrolase	Myricetin	3,4,5-trihydroxyphenylacetic acid; myricetin-3′-*O*-sulfate	Anti-inflammatory↑	Zhang S. et al. (2019) and Káňová et al. (2020)	
	*B. longum* R0175	Ring-cleavage; demethylation	Phloretin hydrolase; demethylase	Hesperidin	3-(3′-hydroxyphenyl) propionic acid; 3-(phenyl) propionic acid	Bioavailability↑	Pereira-Caro et al. (2018)	
	*Eggerthella lenta;* *Flavonifractor plautii*	C-ring cleavage	NA	(−)-epicatechin; (+)-catechin	1-(3,4-dihydroxyphenyl)-3-(2,4,6-trihydroxyphenyl) propan-2-ol; δ-(31,41-dihydroxyphenyl)-γ-valerolactone; δ-(31,41-dihydroxyphenyl)-γ-valeric acid	Bioavailability↑	Ozdal et al. (2016)	
	Human gut microbes	C-ring cleavage A-ring fission dehydroxylation	NA	Anthocyanidin	Protocatechuic acid; syringic acid; vanillic acid; phloroglucinol aldehyde	Bioavailability↑	Aura et al. (2005)	
	Human gut microbes	C-ring cleavage A-ring fission dehydroxylation, etc	Tannase	Procyanidin B2 and A2	2-(3,4-dihydroxyphenyl) acetic acid; 5-(3,4-dihydroxyphenyl)-γ-valerolactone; benzoic acid	Anti-oxidant↑	Stoupi et al. (2010), Ou et al. (2014) and Le Bourvellec et al. (2019)
Alkaloids	Human gut microbes	Ring fission	NA	Strychnine *N*-oxide	Strychnine; 16-hydroxystrychnine	Toxicity↓	el-Mekkawy et al. (1993)
Lignins	*Eggerthella lenta*	Ring cleavage	Benzyl ether reductase	Pinoresinol	Lariciresinol; secoisolariciresinol	Anti-apoptosis↑	Bess et al. (2020)s and Xiao et al. (2021)
Aliphatics	Human gut microbes	Dehydroxylation	NA	Myristic acid	Quercetin-3-*O*-rhamnoside; quercetin; aglycone myricetin	Anti-oxidant↑	Du et al. (2014)

This section summarizes the biotransformation of gut microbiota-mediated natural products from a single reaction. However, some limitations are observed. Firstly, considering the complexity of gut microbes and the diversity of gut microbial enzymes, natural products undergo complex transformations in the intestinal tract. A single reaction can only describe a certain process of metabolism. Therapy can be optimized by activating/inhibiting this process. In addition, considering that gut microbes contain various potentially multifunctional enzymes, more biotransformation reactions underplayed by natural products can be expected from gut microbes. To elucidate how gut microbial metabolism affects human health, researchers should link the functions of interest to genes and enzymes. A deep understanding of the gene sequences of functional enzymes allows organisms with similar sequences to be assigned the same biological activity. Moreover, in addition to the regulation of gut microbes on the disposal of natural products, the regulation of natural products on gut microecology is important as a potential mechanism of efficacy.

## Biotransformation contributions to mining the active substance and mechanism

The increasing research about gut microbiota gradually reveals the relationship between high pharmacological action and low oral availability of most natural products. Most glycosides have complex parent structures and are difficult to be absorbed by the intestine cells, thus limiting their tissue-specific bio-accessibility. These compounds are transformed into small molecule metabolites/unique metabolites through degradation reactions that are dependent on microbial/gut microbial enzymes and thus have a wide range of effects on the host (Wardman et al., 2022). Gut microbes also act on dietary phenolics to produce functional metabolites that contribute to host health (Loo et al., 2020).

**Figure 5:**
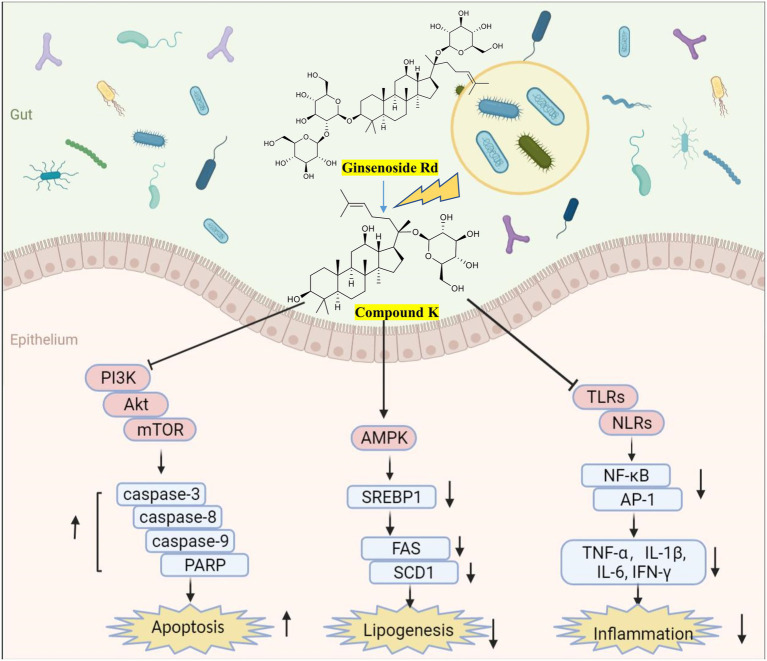
Biotransformation of ginsenosides (Kim et al., 2008) and efficacy of metabolite compound K. Created with BioRender.com.

Importantly, the biotransformation by gut microbes facilitates the therapeutic effects of natural products. The typical metabolization model of ginsenosides to compound K (CK) has been widely reported (Figure 5), with enhanced anti-tumor, anti-inflammatory, and lipid-lowering effects (Kim et al., 2013; Kim, 2018). At 50 μM, CK inhibits the growth of glioblastoma cells by upregulating caspase-3-, caspase-8-, caspase-9- and cAMP-dependent protein kinases (Lee et al., 2017); At 20 μM, CK reduces hepatic lipid accumulation in human hepatocellular carcinoma cells by activating AMPK (Zhang et al., 2022); CK attenuates macrophage inflammation and foam cell formation *via* autophagy induction and by modulating NF-κB, p38 and JNK/MAPK signaling (Lu et al., 2020). The bioavailability of curcumin metabolites is dependent on the microbiota dependent (Hassaninasab et al., 2011). For instance, DMC increases PPARγ expression, resulting in autophagy and NF-κB inhibition and subsequently inhibiting LPS-induced inflammation (Tang et al., 2021). DMC mitigates inflammatory responses *in vivo* and *in vitro* by inhibiting the secretion of inflammatory factors and activation of MAPK and NF-κB pathways (Lu et al., 2022). The chemical stability of DMC increases because of the absence of the methoxyl group in their prototype benzene ring structure, thus explaining the strong beneficial effects of curcumin (Burapan et al., 2017a). Notably, urolithin A (UA), a natural compound that is produced by gut microbes from ingested ellagitannins and ellagic acid, has significant anti-inflammatory and neuroprotective effects. At 1 μM, UA is sufficient for the decreased production of TNF-α and MCP-1 and the inactivation of TLR3/TRIF signaling in poly (I:C)-induced RAW264.7 cells (Huang et al., 2022). UA improves systemic insulin sensitivity and reduces liver IL-1β levels in high-fat diet mice (Toney et al., 2019). UA ameliorates cognitive impairment in APP/PS1 mice and inhibits neuroinflammation by decreasing the levels of IL-6, IL-1β, and TNF-α in the cortex and hippocampus (Gong et al., 2019). These studies highlight the importance of identifying natural products-microbial metabolism. Moreover, many *in vitro* pharmacological activity measurements should be performed in conjunction with microbial metabolites, which actually interact with biochemical receptors *in vivo*.

The composition, structure, function, and metabolites of gut microbes have become potential targets for natural products to exert beneficial effects and reduce toxicity as well. For instance, gut microbes can catalyze the ester bond hydrolysis of C-8 and C-14 of DDAs through CEs or catalyze the ester exchange of C-8 to produce less toxic MDAs (Zhang et al., 2015; Ding et al., 2019). The digoxin-reducing type strains of *E. lenta* contain cardiac glycoside reductase that can reduce the α and β-unsaturated lactone on the digoxin ring and metabolize it into dihydrodigoxin with less activity, thereby inhibiting its possible cardiotoxicity (Kumar et al., 2018). However, this ability is limited, and 50% of digoxin can be inactivated by gut microbial transformation (Lu et al., 2014). Cardiac glycoside reductase may be an effective biomarker for digoxin inactivation, and its expression can be inhibited by arginine (Haiser et al., 2013). Therefore, diet could explain the inter-individual variations in digoxin reduction and may modulate microbial metabolic activity *in vivo*. By contrast, toxic compounds can be produced by gut microbes. Cycasin is hydrolyzed into carcinogenicity diazomethane under the action of β-glucosidase from gut microbes (Goldin, 1990). Therefore, small molecule inhibitors of microbial gut enzymes should be developed to play a regulatory role in specific transformation in this complex habitat. The toxicity difference between metabolites transformed by gut microbiota and precursor substances is worthy of further study. Moreover, excessive drugs may cause imbalance and adverse reactions in gut microbes (Lindell et al., 2022), and the effects of different doses of natural products on gut microbes and metabolism need further investigation.

## Multivariate technologies for studying biotransformation

Considering that gut microbes can increase the host’s complex and variable response to drugs/natural products, this process is of great interest to researchers. Research on biotransformation is mainly conducted *via in vitro* approach (Sousa et al., 2008) as follows: (1) Intestinal fluid transformation. The large-scale preparation of transformed products can be realized by intestinal fluid biotransformation; (2) Incubation with a sample of the host microbiota. The type and quantity of prototype drugs and metabolites can be detected using the method. It has the advantage of accurate representation of the entire gut microbiome of the individual; (3) Incubation of representative strains. This method affords high-throughput potential, which is valuable for large-scale drug studies and contributes to the industrial production of beneficial metabolites. In addition, organ-on-a-chip microphysiological systems (Ashammakhi et al., 2020), gastrointestinal organoids (Singh et al., 2020), and various predictive/computational tools (Machado et al., 2018; Chowdhury and Fong, 2020) may help improve our understanding of microbial metabolism in the future.

In addition, the relationships between natural product metabolism and gut microbes have been studied in animal models, and the results can be used to investigate the distribution and form of metabolites (Yoshisue et al., 2000). Germ-free/antibiotic-treated animals with conventional animals have been compared to prove the key roles of gut microbes on natural product metabolism. The limitation of this method is that inherent gastrointestinal and microbiological differences exist between humans and rodents (Nguyen et al., 2015). Detailed microbiota and metabolite analysis of feces collected from subjects in clinical trials can comprehensively reflect the metabolic process of natural products *in vivo* and be used to explain individual differences. In addition, the application of sequencing technology needs to be increased to study the microbial transcriptional activity and metabolic profile. By using the single-cell method, the physiological structure of gut microbes can be characterized to determine their metabolic activity (Zheng et al., 2022). Metatranscriptomics (RNA-Seq) allows the direct analysis of gene expression profiles of microorganisms with strong metabolic activity in the human gut (Berlinberg et al., 2022). The combination of single-cell methods, metatranscriptomics, and metagenomics has been used to identify and characterize the active subsets of gut microbiota and determine their metabolic responses to natural products.

## Conclusions and future remarks

The gut microbiota is a reservoir of genes that encode various metabolic enzymes (Flint et al., 2012). The activation of biological activities and potential health benefits of most natural products (e.g., flavonoids, alkaloids, and lignin) are extremely dependent on gut microbes as a substrate-machining factory (Braune and Blaut, 2016; Seyed Hameed et al., 2020; Plamada and Vodnar, 2021). Much research effort has been devoted to understanding how microbes uniquely modify natural products and the effects of these metabolites on host health (Luca et al., 2020; Shabbir et al., 2021). The following conclusions have been made: (1) gut microbes can transform natural products (Xie et al., 2020); (2) natural products can regulate the composition and abundance of gut microbes (Saccon et al., 2021); and (3) gut microbes can mediate the multi-component synergy of natural products (Feng et al., 2019). Although high-throughput methods are being developed to help people understand the importance of the gut microbiome in the metabolism of natural products, microbial metabolism-based screening has not been adopted as part of the drug development process, because its mechanism remains unclear (Zimmermann et al., 2019). Moreover, the great plasticity and interindividual differences of gut microbes are notable (Vujkovic-Cvijin et al., 2020). Therefore, researchers need to improve the understanding of the physiological, chemical, and microbial contributions of gut microbes to the metabolism of natural products to help in explaining the individual differences in natural product responses and provide support for personalized treatment (Kolodziejczyk et al., 2019; Javdan et al., 2020). Most of the data in the present study were obtained independently of the clinic, but clinical trials are already underway, and the results will influence clinical practice in the foreseeable future.

Increasing studies on the mechanism of how to exert the curative effect, the application of fecal transplantation, specific bacterial transplantation, and animal models will help in clarifying the role of gut microbes. Nevertheless, standardization of operation, reproducibility of experimental results, and variation between species and individuals greatly reduce the authenticity and stability of the research, and a standard and scientific operating procedure remain to be put forward. Thus, confirming the symbolic functional extremely involved in biotransformation and its material basis will help in exploring the mechanism of natural products in the treatment of diseases and explaining the treatment mode of indirect interaction between natural products with low bioavailability and gut microbiota.

## Author contributions

YZ contributed to the data collection and preparation of the original draft. XZ, JY, and CS provided brief article ideas and language modifications. XZ and XW supervised and revised the manuscripts. All authors contributed to the article and approved the submitted version.

## Funding

This work was supported by the National Natural Science Foundation of China under grant no. 81873104, 81830112, and 82192914.

## Conflict of interest

The authors declare that the research was conducted in the absence of any commercial or financial relationships that could be construed as a potential conflict of interest.

## Publisher’s note

All claims expressed in this article are solely those of the authors and do not necessarily represent those of their affiliated organizations, or those of the publisher, the editors and the reviewers. Any product that may be evaluated in this article, or claim that may be made by its manufacturer, is not guaranteed or endorsed by the publisher.

## Footnotes

^1^ https://www.nature.com/subjects/natural-products

## Abbreviations

DHC: dihydrocurcumin
THC: tetrahydrocurcumin
CurA: NADPH-dependent curcumin/dihydrocurcumin reductase
CHA: chlorogenic acid
CA: caffeic acid
GL: glycyrrhizin
18β-GA: 18β-glycyrrhetinic acid
PM-I: paeonimetabolin-I
GAMG: 18β-glycyrrhetinic acid-3-*O*-β-*D*-glucuronic acid
SGG: *Streptococcus gallolyticus* subsp. *Gallolyticus*
DDAs: diester diterpenoid alkaloids
MDAs: monoester diterpene alkaloids
DHENL: (−)-dihydroxyenterolactone
3′-DMAG: 3′-desmethylarctigenin
BBR: Berberine
dhBBR: dihydroberberine
DMC: demethoxycurcumin
bDMC: bis-demethoxycurcumin
CK: compound K
UA: Urolitin A
